# Constructing high-performance electrode materials using core–shell ZnCo_2_O_4_@PPy nanowires for hybrid batteries and water splitting†

**DOI:** 10.1039/d0ra05177b

**Published:** 2020-07-29

**Authors:** Xiaoyun Liu, Qian Li, Yanli Qin, Yueqiu Jiang

**Affiliations:** School of Science, Shenyang Ligong University Shenyang 110159 P. R. China liuxy@imr.ac.cn; Department of Development and Planning, Shenyang Ligong University Shenyang 110159 P. R. China yueqiujiang@sylu.edu.cn

## Abstract

Heterogeneity can be used as a promising method to improve the electrochemical performance of electrode materials; thus, ZnCo_2_O_4_@PPy samples were prepared using a facile hydrothermal route and an electrochemical deposition process. The as-prepared products possess a specific capacitance of 605 C g^−1^ at a current density of 1 A g^−1^. The asymmetric supercapacitor (ASC) possesses an energy density of 141.3 W h kg^−1^ at a power density of 2700.5 W kg^−1^ and capacity retention of 88.1% after 10 000 cycles, indicating its promising potential for energy devices. ZnCo_2_O_4_@PPy-50 exhibited an excellent OER performance and outstanding HER performance in alkaline media. As an advanced bifunctional electrocatalyst for overall water splitting, a voltage of 1.61 V at a current density of 50 mA cm^−2^ outperforms the majority of noble-metal-free electrocatalysts.

## Abstract 2

Heterogeneity can be used as a promising method to improve the electrochemical performance of electrode materials; thus, ZnCo_2_O_4_@PPy samples were prepared using a facile hydrothermal route and an electrochemical deposition process.

## Graphical abstract

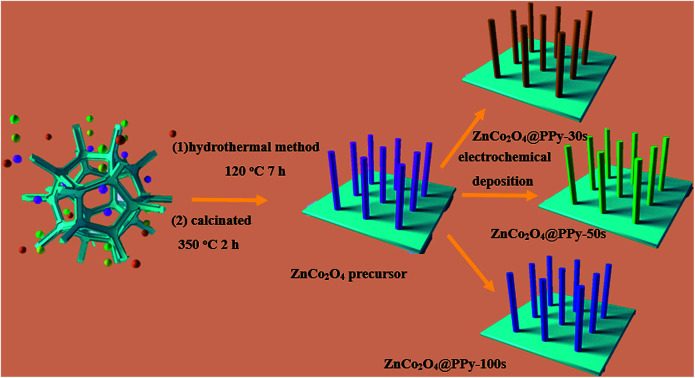

## 1. Introduction

Our rapidly developing economic society requires a technological evolution to construct high-efficiency energy-storage devices for future renewable power tools.^1–5^ Supercapacitors exhibit excellent cycling, fast-charging abilities, and small sizes, which makes them the most promising candidates for next generation power devices.^6–8^ However, for practical applications, the low power density limits their rapid development.^9–12^ At the same time, electrode materials play a crucial role in high-performance devices. Thus, the design and selection of ideal electrode materials can effectively solve this problem.^13^ In addition, electrocatalytic water splitting has been regarded as the most hopeful method to generate high-purity hydrogen.^14,15^ However, the efficiency of water splitting is restricted by the kinetically sluggish oxygen evolution reaction (OER).^16,17^ Therefore, the construction of efficient and economical electrode materials is the key to improving the device performance and fabricating highly active catalysts.

Owing to their rich redox active sites and large specific surface areas, various transition metal oxides, including ZnO, SnO_2_, Co_3_O_4_, and ZnCo_2_O_4_ materials, have been widely investigated.^18–21^ Among them, ZnCo_2_O_4_ electrodes stand out because of their high theoretical capacitance and a high number of active sites.^22,23^ For example, Pail *et al.* fabricated a rGO–ZnCo_2_O_4_ nanocomposite through a hydrothermal method, which possesses a specific capacitance of 500.8 F g^−1^ at a current density of 1 A g^−1^.^24^ The Kamble group synthesized a ZnCo_2_O_4_ thin film electrode, which presents a specific capacitance of 127.8 F g^−1^ at a current density of 1 A g^−1^ and 80.6% capacity retention after 3000 cycles.^25^ Su and coworkers researched flower-like ZnCo_2_O_4_ with a specific capacitance of 384 F g^−1^ at a current density of 1 A g^−1^.^26^ Choi *et al.* reported a ZnCo_2_O_4_ electrode, which was fabricated through simple methods. The samples can be directly utilized as electrode materials and show excellent OER performances.^27^ However, the single-crystalline ZnCo_2_O_4_ electrodes possess poor cycle stability. An effective strategy for improving this is compounding with conductive polymers. As a typical conjugated polymer, PPy is considered to be a very promising electrode material due to its high electrical conductivity. At the same time, reports indicate that combining PPy with metal compounds can greatly improve the specific capacitance and cycle performance as well as decrease the overpotential. This can be attributed to PPy promoting electron transport and reaction internal resistance.

In this work, we synthesized novel ZnCo_2_O_4_@PPy-50 nanostructures directly grown on Ni foam using a hydrothermal method and an electrochemical deposition process. The ZnCo_2_O_4_@PPy electrode materials show a capacitance of 605 C g^−1^ at a current density of 1 A g^−1^. When used as anode materials, the as-fabricated device exhibits an energy density of 141.3 W h kg^−1^ at a power density of 2700.5 W kg^−1^ and a capacity retention of 88.1% after 10 000 cycles. As electrocatalysts, the ZnCo_2_O_4_@PPy-50 samples show a low overpotential of 240 mV at 50 mA cm^−2^ and a low cell voltage of overall water splitting.

## 2. Experimental details

In the experiments, Ni foam (4 × 4 cm^2^) was immersed in a 1.0 M HCl solution for 30 min. Next, the Ni foam was repeatedly cleaned with ethanol and deionized water and then dried overnight.

### 2.1 Synthesis of the ZnCo_2_O_4_ nanowires

The ZnCo_2_O_4_ nanowires were fabricated on Ni foam *via* a facile hydrothermal method. 2 mmol Zn(NO_3_)_2_·6H_2_O, 4 mmol Co(NO_3_)_2_·6H_2_O, 3 mM NH_4_F, and 6 mM urea were dissolved in 60 mL deionized water and magnetically stirred for 30 min. Then, the pre-treated Ni foam was placed in a uniform solution and kept at 140 °C for 6 h. After cooling to room temperature, the ZnCo_2_O_4_ precursor was washed with deionized water and ethanol and dried at 60 °C overnight. Afterwards, the sample was calcinated at 350 °C for 2 h. The average mass loading of the sample was 1.9 mg cm^−2^.

### 2.2 Synthesis of the ZnCo_2_O_4_@PPy composite structures

The PPy nanofilm was fabricated on ZnCo_2_O_4_ nanowire surfaces by the electrochemical deposition method. For the cathodic deposition, a solution of 1 mL Py and 3 g *p*-toluenesulfonic acid was used as the electrolyte. The deposition was carried out in a three-electrode system, consisting of a piece of Ni foam with ZnCo_2_O_4_ as the working electrode, a Pt foil as the counter electrode, and Ag/AgCl as the reference electrode. The deposition was carried out at a constant potential of 0.95 V for 50 s at room temperature. The final samples were washed with deionized water and dried at 60 °C. The average mass loading was 2.0 mg cm^−2^.

### 2.3 Structure characterization

The morphology and crystal structure of the as-prepared samples were studied by X-ray powder diffraction (XRD) using a Shimadzu-7000 with Cu Kα radiation, X-ray photoelectron spectroscopy (XPS) analysis utilizing an ESCALAB 250 with an Al Kα source, and Fourier transform infrared spectroscopy (FTIR, 4000–500 cm^−1^). Scanning electron microscopy (SEM) using a JSM-7001F microscope and transmission electron microscopy (TEM) using JEM-2100 PLUS were also performed. The surface area of the samples was investigated through N_2_ absorption and desorption isotherms.

### 2.4 Electrochemical measurements

The electrochemical characteristics of the electrode materials were studied using a CHI660E electrochemical workstation (Chenhua, Shanghai). Cyclic voltammetry (CV) curves, galvanostatic charge–discharge (GCD) tests and electrochemical impedance spectroscopy (EIS) results were measured in a three-electrode system in 3.0 M KOH solutions. The as-produced samples, Pt foil, and Hg/HgO were used as the working electrode, counter electrode, and reference, respectively.

### 2.5 Assembly of asymmetric supercapacitors (ASCs)

Asymmetric supercapacitors were constructed using the ZnCo_2_O_4_@PPy-50 samples as the cathode and active carbon (AC) as the anode. The specific capacitance (*C*_s_) of the electrode materials were obtained using discharge times (Δ*t*) according to the following equation:

1 *C*_s_ = *I*Δ*t*/*m*Δ*V*

where *I* is the current density, *m* represents the mass of the electrode, and Δ*V* denotes the potential windows.

2 *E* = 1/2*CV*^2^

3 *P* = 3600*E*/*t*

where *E* is the energy density, *C* stands for the specific capacitance, *P* represents the power density, and *V* refers to the voltage window.

### 2.6 Electrocatalytic performance

Linear sweep voltammetry (LSV) polarization curves and long-term durability measurements were obtained on a CHI 660E electrochemical workstation (Shanghai Chenhua) with Ag/AgCl as the reference electrode and Pt foil as the counter electrode, and the as-prepared samples as the working electrode in 1.0 M KOH electrolyte.

## 3. Results and discussion

**Fig. 1:**
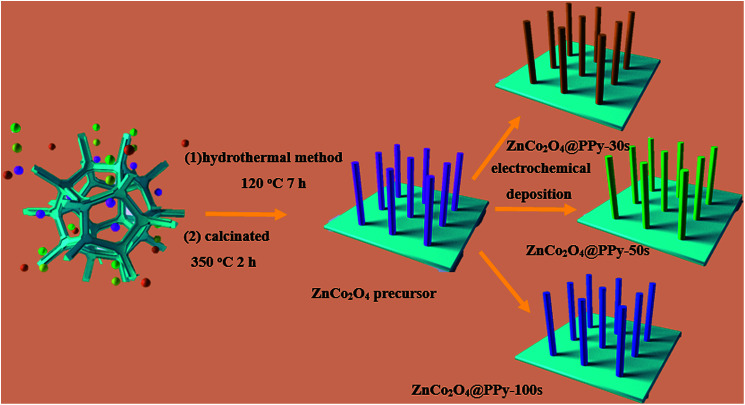
Schematic illustration of the fabrication of ZnCo_2_O_4_@PPy samples.

The fabrication process of the positive electrode is schematically illustrated in Fig. 1. Firstly, Ni foam uniformly coated with ZnCo_2_O_4_ samples is obtained using a facile hydrothermal method. Subsequently, by using an electrochemical deposition method, which employs ZnCo_2_O_4_ as the precursor under different conditions, the positive electrodes can be immediately obtained.

**Fig. 2:**
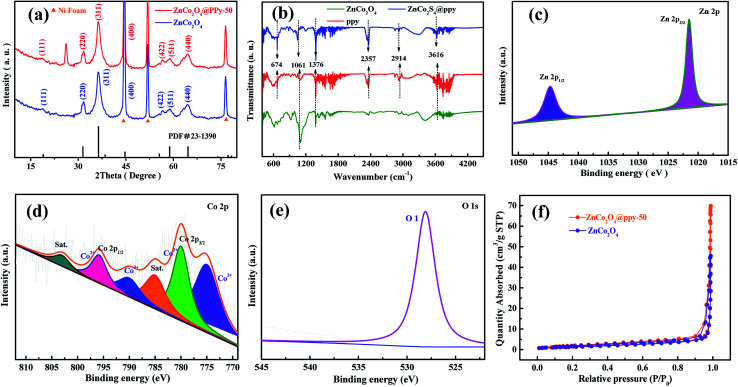
(a) XRD patterns of the as-produced samples (b) FTIR spectra (c) Zn 2p XPS spectra of the composites (d) Co 2p (e) O 1s (f) N_2_ adsorption/desorption isotherms.

Concerning the composition of the as-prepared materials, typical XRD patterns of the ZnCo_2_O_4_ and ZnCo_2_O_4_@PPy samples are shown in Fig. 2a, in which the highest peaks are from the Ni foam (2*θ* = 44.6°, 51.8°, and 76.6°). In the blue curves, the main peaks at 2*θ* values of 31.6°, 36.4°, 44.6°, 59.1°, and 64.5° can be indexed to the (220), (311), (400), (511), and (440) planes of the cubic spinel ZnCo_2_O_4_ phase (PDF no. 23-1390). In the XRD pattern of the composite structure, the diffraction peaks located at the same peak position may be assigned to the ZnCo_2_O_4_ phase. In the XRD pattern of the composite material, a clear peak appears at about 26°, proving that PPy successfully coated the surface of ZnCo_2_O_4_. FTIR spectroscopy was utilized to further study the distinct structures of the ZnCo_2_O_4_, PPy, and ZnCo_2_O_4_@PPy materials (Fig. 2b). The peak situated at 674 cm^−1^ is attributed to the Zn–O vibrations of the ZnCo_2_O_4_ electrode material. The peaks located at 1061 cm^−1^ correspond to the C–H and C–N vibration of PPy.^28^ The strong peak at 3616 cm^−1^ is attributed to absorbed water. In addition, the peaks at 2357 cm^−1^ and 2914 cm^−1^ come from N–H bonds in the aromatic amines. In the FITR spectra of the ZnCo_2_O_4_@PPy materials, all of the peaks are present, which proves that PPy is successfully coated on the surface of the ZnCo_2_O_4_ samples.

The XPS pattern of the ZnCo_2_O_4_@PPy-50 material was measured to comprehend the surface chemical states. The C 1s XPS pattern is shown in Fig. S1.† In Fig. 2c, the Zn 2p spectrum exhibits two strong peaks at 1044.60 eV and 1021.60 eV, which are attributed to the binding energy of Zn 2p_1/2_ and Zn 2p_3/2_, respectively, indicating the existence of Zn^2+^ in the ZnCo_2_O_4_@PPy-50 composite materials. In Fig. 2d, the Co 2p spectrum contains four spin–orbit components, Co 2p_3/2_ and Co 2p_1/2_ and two satellites. The four peaks are at 774.9 eV, 780.1 eV, 790.2 eV, and 795.8 eV, respectively. The strongest splitting peak value is about 15.3 eV, which is attributed to the existence of Co^3+^ and Co^2+^.^29^ As shown in Fig. 2e, the O 1s XPS spectrum of the composite materials consist of the O 1s peak (528.1 eV), which is attributed to the metal–oxygen bonds. Fig. 2f exhibits the N_2_ isotherms of the ZnCo_2_O_4_ and ZnCo_2_O_4_@PPy-50 samples. The IV isotherm (ZnCo_2_O_4_@PPy-50 electrode material) is presented at a relative pressure between 0 and 1.0, which indicates the presence of the mesoporous structure. The BET surface areas of the ZnCo_2_O_4_ and ZnCo_2_O_4_@PPy-50 samples are calculated to be 45 and 69 m^2^ g^−1^, respectively. It can be found that the ZnCo_2_O_4_@PPy-50 electrodes possess a larger surface area than the ZnCo_2_O_4_ electrode materials.

**Fig. 3:**
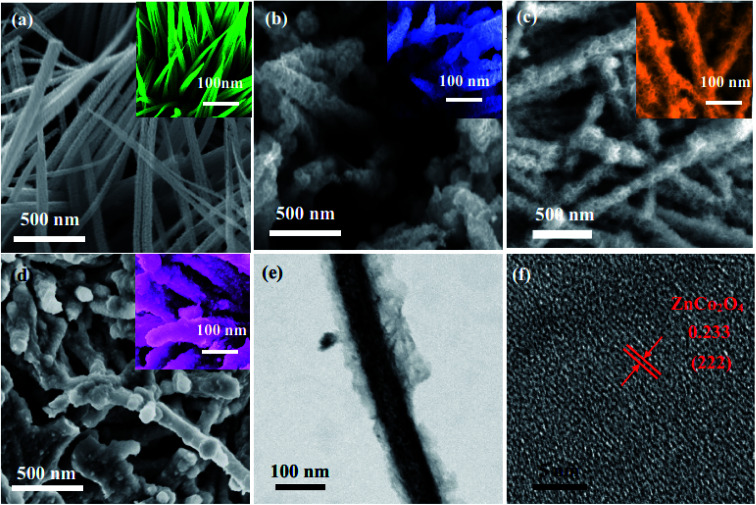
SEM images of (a) ZnCo_2_O_4_ (b) ZnCo_2_O_4_@PPy-20 (c) ZnCo_2_O_4_@PPy-50, and (d) the ZnCo_2_O_4_@PPy-100 samples, TEM images of the ZnCo_2_O_4_@PPy-50 electrode materials (e) low magnification (f) HRTEM images.

The morphologies of the electrode materials are observed by SEM. As shown in Fig. 3a, the ZnCo_2_O_4_ samples possess wire-like shapes. From the high magnification SEM images, the diameter of the nanowires is approximately 20 nm. In Fig. 3b, it can be seen that PPy is covered on the surface of the ZnCo_2_O_4_ electrode materials. After an electrochemical deposition process, the morphologies of the ZnCo_2_O_4_@PPy-20 samples are more rough and uneven than the ZnCo_2_O_4_ products. The average diameter of ZnCo_2_O_4_@PPy-50 is around 25 nm (Fig. 3c), which leads to a large surface area with an extensive number of active sites. This structure is more conducive to the transmission of electrons. The structural information of the ZnCo_2_O_4_@PPy-50 samples was further obtained by TEM (Fig. 3e and f). It is consistent with the XRD results. As shown in Fig. 3f, the ultra-thin PPy is evenly coated on the surface of the ZnCo_2_O_4_ samples. The HRTEM image (Fig. 2i) exhibits interplanar spacings of 0.233 nm, corresponding to the (222) plane of the cubic ZnCo_2_O_4_ structures.

**Fig. 4:**
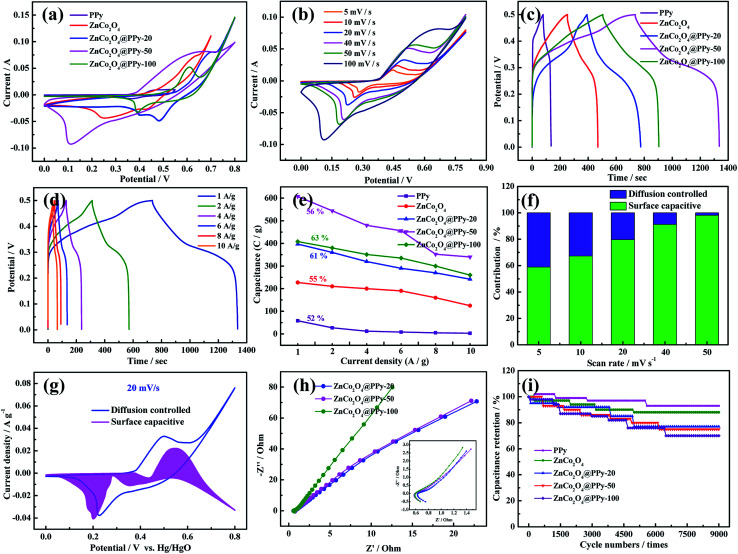
(a) CV curves of PPy, ZnCo_2_O_4_, ZnCo_2_O_4_@PPy-50, and the ZnCo_2_O_4_@PPy-100 electrode materials at 100 mV s^−1^ (b) CV curves of the ZnCo_2_O_4_@PPy-50 sample at different scanning rates (c) GCD curves at a current density of 1 A g^−1^ (d) GCD curves of the ZnCo_2_O_4_@PPy-50 sample at different current densities (e) trends in the specific capacitance values with current density for the as-prepared samples (f) contribution ratio between the capacitance and diffusion-limited capacitance (g) capacitive and diffusion contributions at a scan rate of 5 mV s^−1^ (h) Nyquist plots (i) cycling performance.

In order to compare the electrochemical performance of the three-electrode materials, CV, GCD, and EIS curves were measured in a 3.0 M KOH electrolyte using a standard three-electrode system with a Pt foil counter electrode, a Hg/HgO reference electrode, and the as-prepared samples as the working electrode. For comparison, the electrode materials were tested under the same conditions. In Fig. 4a, the ZnCo_2_O_4_@PPy-50 electrode materials show a larger area than the other four-electrode materials at a scan rate of 100 mV s^−1^, suggesting that synergy can significantly increase the capacitance of the electrode.^30^ As seen in Fig. 4b, the CV curves possess obvious redox peaks. As the sweep speed increases, the area of the curve increases, demonstrating that the electrode materials possess good reversibility. In Fig. 4c, the ZnCo_2_O_4_@PPy-50 electrode exhibits a longer discharge time than the other four materials. The specific capacitances of the composite samples are significantly increased, reaching 605 C g^−1^ at 1 A g^−1^. Fig. 4d presents the GCD curves of the ZnCo_2_O_4_@PPy-50 electrodes. The discharge time of the samples decreases with increasing current density.

We calculated the areal specific capacitance of the test electrodes at different current densities from 1 A g^−1^ to 10 A g^−1^ to evaluate their charge/discharge rate performance, as shown in Fig. 4e. It can be perceived that the ZnCo_2_O_4_@PPy-50 electrode still maintains 56% of the capacitance value at 10 A g^−1^. The CV curve is an efficient means to study the reaction kinetics of the as-prepared materials:^31^

4 *i* = *k*_1_*v* + *k*_2_*v*^1/2^

The *k*_1_ and *k*_2_ values can be obtained *via* the CV curves. The surface capacitive and diffusion-controlled contributions to the total stored charges of the electrodes are shown in Fig. 4f. For the as-prepared electrode material, the diffusion controlled contribution gradually reduces with increasing scan rate, resulting in a decrease in the total capacitance at high scan rates. The capacitive contribution of the ZnCo_2_O_4_@PPy-50 electrode material (Fig. 4g) is 79% at a scan rate of 20 mV s^−1^. The capacitive and diffusion contributions at a scan rate of 5, 10, 40, and 50 mV s^−1^ are shown in Fig. S2.† The capacitive contribution of the sample is 98% at a scan rate of 50 mV s^−1^. The EIS analysis of the as-prepared products is shown in Fig. 4h and the ZnCo_2_O_4_@PPy-50 electrode material exhibits the smallest semicircle and maximum slope when compared with the ZnCo_2_O_4_@PPy-20 and ZnCo_2_O_4_@PPy-100 materials, revealing the faster diffusion rate of the electrons and better conductivity. The intercepts of the ZnCo_2_O_4_@PPy-20, ZnCo_2_O_4_@PPy-50, and ZnCo_2_O_4_@PPy-100 electrode materials with the *X* axis are 0.64, 0.52, and 0.59 Ω, respectively. This means that the ZnCo_2_O_4_@PPy-50 electrode material possesses the lowest internal resistance. Fig. 4i presents the capacitance retention of the as-prepared samples as a function of the cycle number at 10 A g^−1^. The capacitance retention of the ZnCo_2_O_4_@PPy-50 electrode material is 93.5%.

**Fig. 5:**
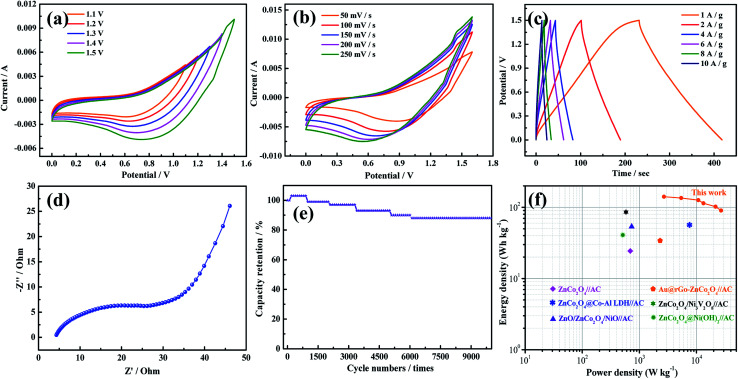
Electrochemical characterizations of the ASC (a) CV curves of the ZnCo_2_O_4_@PPy//AC ASC at different voltage windows (b) CV curves of the ZnCo_2_O_4_@PPy//AC ASC at different scan rates (c) GCD curves (d) Nyquist plots (e) cycling performance (f) Ragone plots.

To evaluate the efficiency of the asymmetric supercapacitors (ASCs), the CV curves of the ASCs at different voltage windows were obtained and show that the ZnCo_2_O_4_@PPy-50//AC ASC has a high operating cell voltage of up to 1.5 V (Fig. 5a). The curve was measured at the scan rate of 100 mV s^−1^. Fig. 5b shows the CV curves at different scan rates from 5 mV s^−1^ to 100 mV s^−1^. With an increasing sweep rate, the shape of the curve remains unchanged, indicating that the device displays an ideal capacitance performance. To further study the capacitive behavior of the devices, the discharge curves between 0 and 1.5 V at different current densities are illustrated in Fig. 5c. The discharge time of the device is 188 s, indicating that the ZnCo_2_O_4_@PPy-50//AC device exhibits superior electrochemical performance. The EIS analyses of the devices are shown in Fig. 5d. It is obviously found that the device shows a low bulk resistance and the internal resistance of the device is 0.65 Ω. Fig. 5e shows that the capacitance retention of the device is 88.1% after 10 000 cycles. Based on the discharge curves at different current densities, the power densities and energy densities of the device were calculated using Ragone plots (Fig. 5f). The ASC delivers a maximum energy density of 141.3 W h kg^−1^ at a power density of 2700.5 W kg^−1^, which is higher than that of the other reported devices.^21,24,32–35^

**Fig. 6:**
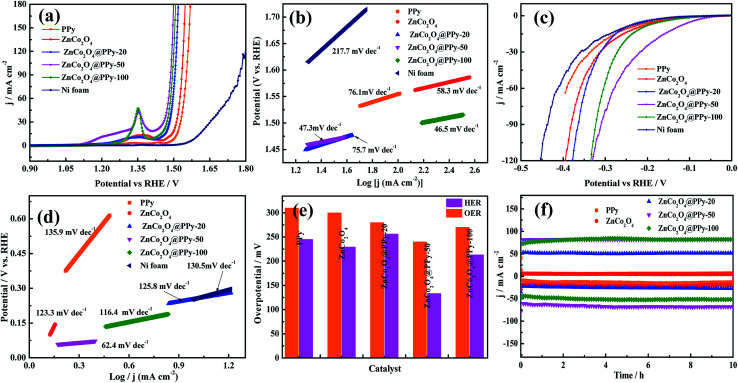
(a) Polarization curves (OER) of the as-prepared samples at a scan rate of 2 mV s^−1^ (b) Tafel plots for the OER performances (c) polarization curves (HER) of the as-prepared samples with a scan rate of 5 mV s^−1^ (d) Tafel plots of the HER performances (e) required overpotential of the different electrocatalysts for OER and HER (f) chronopotentiometry curves.

The OER performance and HER activity of the as-prepared samples were tested using a three-electrode system in a 1.0 M KOH electrolyte. Before the measurement, the electrode materials were activated 50 times through the cyclic voltammetric curves. The OER performance was studied by LSV curves at a scan rate of 1 mV s^−1^. As shown in Fig. 6a, in comparison with Ni foam, PPy, ZnCo_2_O_4_, ZnCo_2_O_4_@PPy-20, and ZnCo_2_O_4_@PPy-100, the LSV curves of the ZnCo_2_O_4_@PPy-50 (*η*_50_ = 240 mV) sample possess the highest current under a certain applied voltage. To further research the OER performance, Tafel slopes were obtained and are exhibited in Fig. 6b. The values of the Tafel slopes were calculated from the LSV curves. The Tafel slope of ZnCo_2_O_4_@PPy-50 is 46.5 mV dec^−1^, which is smaller than that of Ni foam (217.7 mV dec^−1^), PPy (76.1 mV dec^−1^), ZnCo_2_O_4_ (58.3 mV dec^−1^), ZnCo_2_O_4_@PPy-20 (75.7 mV dec^−1^), and ZnCo_2_O_4_@PPy-100 materials (47.3 mV dec^−1^). The smaller value demonstrates that ZnCo_2_O_4_@PPy-50 possesses a rapid OER reaction rate.

The HER activity was measured for the same device. The LSV curves (Fig. 6c) were obtained at a scan rate of 5 mV s^−1^. The ZnCo_2_O_4_@PPy-50 material exhibits lowest overpotential (*η*_10_ = 133 mV) at a current density of 10 mA cm^−2^. As we can observe in Fig. 6d, the value of 62.4 mV dec^−1^ for ZnCo_2_O_4_@PPy-50 is much lower than 130.5 mV dec^−1^ for the Ni foam, 135.9 mV dec^−1^ for PPy, 123.3 mV dec^−1^ for ZnCo_2_O_4_, 125.8 mV dec^−1^ for ZnCo_2_O_4_@PPy-20, and 116.4 mV dec^−1^ for ZnCo_2_O_4_@PPy-100. A lower Tafel slope is more favorable for HER performance. In Fig. 6e, the overpotentials of the as-prepared samples are compared in detail. ZnCo_2_O_4_@PPy-50 shows the smallest overpotential (OER and HER).

**Fig. 7:**
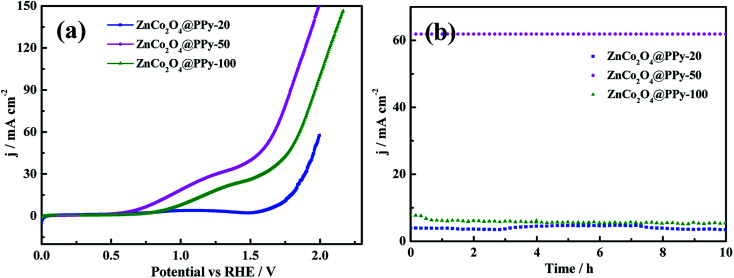
(a) Polarization curves of the overall water splitting at a scan rate of 5 mV s^−1^ (b) durability of the electrode at a current density of 10 mA cm^−2^.

It is important for catalysts to have excellent stability for practical application. The ZnCo_2_O_4_@PPy-50 electrode materials show outstanding stability with no significant decrease after 10 h. As shown in Fig. 7a, the voltage of the overall water splitting electrode, ZnCo_2_O_4_@PPy-50, is 1.61 V at the current density of 50 mA cm^−2^. In Fig. 7b, the current density of the ZnCo_2_O_4_@PPy-50 sample exhibits a steady trend after 10 h.

**Fig. 8:**
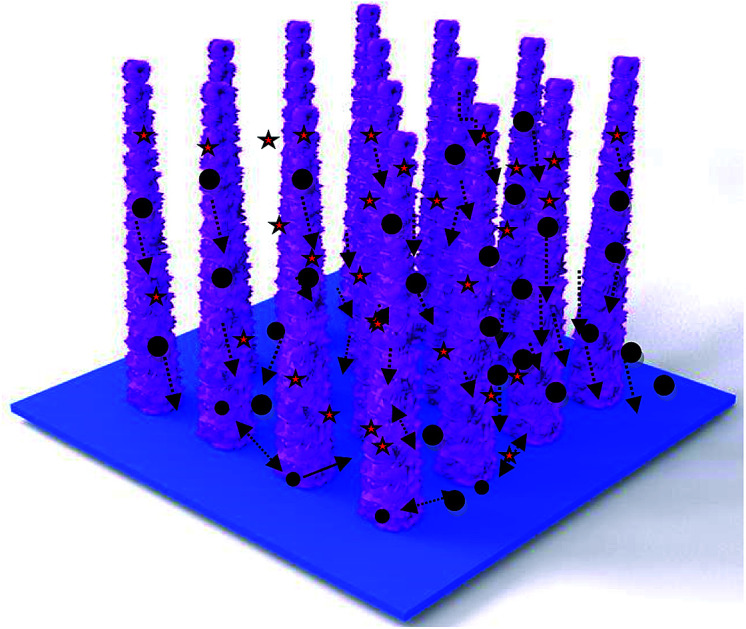
Electrochemical reaction mechanism.

Combined with the electrochemical reaction mechanism (Fig. 8), the excellent electrochemical performance can be attributed to the following: first, the direct growth of ZnCo_2_O_4_ nanowires on Ni foam benefits from sufficient contact between the electrode and current collector. Moreover, the PPy shell can provide high electrical conductivity pathways and accelerate the electron transportation speed. Finally, the synergetic effect between ZnCo_2_O_4_ and PPy also improves the cycling performance of the electrode materials.

## 4. Conclusion

In summary, ZnCo_2_O_4_@PPy-50 electrode material was successfully grown on Ni foam, which possesses a specific capacitance of 605 C g^−1^ at a current density of 1 A g^−1^. In addition, the as-prepared samples retain 93.5% capacitance after 7000 cycles. The ASC possessed an energy density of 141.3 W h kg^−1^ at a power density of 2700.5 W kg^−1^ and capacity retention of 88.1% after 10 000 cycles. ZnCo_2_O_4_@PPy-50 exhibited excellent OER performance and outstanding HER performance in alkaline media. As an advanced bifunctional electrocatalyst for overall water splitting, a voltage of 1.61 V at a current density of 50 mA cm^−2^ outperforms the majority of the noble-metal-free electrocatalysts.

## Conflicts of interest

The authors declare no conflict of interest.

## Supplementary Material

RA-010-D0RA05177B-s001

## References

[cit1] Zhang G. Q., Wu H. B., Hoster H. E., Chan-Park M. B., Lou X. W. (2012). Energy Environ. Sci..

[cit2] Park T., Jang Y., Park J. W., Kim H., Kim S. J. (2020). RSC Adv..

[cit3] Zhao D. P., Dai M. Z., Zhao Y., Liu H. Q., Liu Y., Wu X. (2020). Nano Energy.

[cit4] He X., Wang X. Y., Sun B. N., Wan J. N., Wang Y., He D., Suo H., Zhao C. (2020). RSC Adv..

[cit5] Zhu Y. G., Wang Y., Shi Y. M., Wang J. I., Yang H. Y. (2014). Nanoscale.

[cit6] Zhao S. H., Yu X. B., Chen H. M., Tao K., Hu Y. P., Han L. (2020). RSC Adv..

[cit7] Miller J. R., Simon P. (2008). Electrochemical capacitors for energy management. Science.

[cit8] Dai M. Z., Zhao D. P., Liu H. Q., Tong Y. L., Hu P. F., Wu X. (2020). Mater. Today Energy.

[cit9] Deori K., Ujjain S. K., Sharma R. K., Deka S. (2013). ACS Appl. Mater. Interfaces.

[cit10] Zhao D. P., Dai M. Z., Liu H. Q., Chen K. F., Zhu X. F., Xue D. F., Wu X., Liu J. P. (2019). Adv. Mater. Interfaces.

[cit11] Wang Q. F., Wang X. F., Xu J., Yang X. O., Hou X. J., Chen D., Wang R. M., Shen G. Z. (2014). Nano energy.

[cit12] Chen H. C., Jiang J. J., Zhang L., Xia D. D., Zhao Y. D., Guo D. Q., Qi T., Wan H. Z. (2014). J. Power Sources.

[cit13] Tong Y. L., Qi D. L., Chi B. Q., Zhang W. Q. (2019). Sci. Adv. Mater..

[cit14] Jadhav H. S., Roy A., Chung W. J., Seo J. G. (2017). Electrochim. Acta.

[cit15] Du X., Yang Z., Li Y., Gong Y., Zhao M. (2018). J. Mater. Chem. A.

[cit16] Tong Y. L., Chi B. Q., Qi D. L., Liu X. Y. (2019). Sci. Adv. Mater..

[cit17] Ellis B. L., Knauth P., Djenizian T. (2014). Adv. Mater..

[cit18] Xing L., Dong Y. D., Wu X. (2018). RSC Adv..

[cit19] Tong Y. L., Cheng X. Y., Liu X. Y., Qi D. L., Chi B. Q., Wang Y. F. (2019). J. Nanoelectron. Optoelectron..

[cit20] Zhai T., Wan L., Sun S., Chen Q., Sun J., Xia Q., Xia H. (2017). Adv. Mater..

[cit21] Bhagwan J., Hussain S. K., Yu J. S. (2020). J. Alloys Compd..

[cit22] Kumar Y. A., Kumar K. D., Kim H. J. (2020). Electrochim. Acta.

[cit23] Shi R., Zhang Y. Y., Wang Z. H. (2019). J. Alloys Compd..

[cit24] Pail S. J., Dubal D. P., Lee D. W. (2020). Chem. Eng. J..

[cit25] Kamble G. P., Kashale A. A., Dhanayat S. S., Kolekar S. S., Ghule A. V. (2019). Mater. Sci..

[cit26] Su W., Miao R., Tao B. R., Miao F. J. (2019). Ionics.

[cit27] Kim T. W., Woo M. A., Regis M., Choi K. S. (2014). J. Phys. Chem. Lett..

[cit28] He W. D., Wang C. G., Li H. Q., Deng X. L., Xu X. J., Zhai T. Y. (2017). Adv. Energy Mater..

[cit29] Xiao J. W., Wan L., Yang S. H., Xiao F., Wang S. (2014). Nano Lett..

[cit30] Yi H., Wang H. W., Jing Y. T., Peng T. Q., Wang X. F. (2015). J. Power Sources.

[cit31] Sathiya M., Prakash A. S., Ramesha K., Tarascon J.-M., Shukla A. K. (2011). J. Am. Chem. Soc..

[cit32] Bai X., Cao D. X., Zhang H. G. (2019). Ceram. Int..

[cit33] Huang Y., Feng X. S., Li C., Li Y., Chen X. F., Gao X. G., Chen C., Guang Z. X., Liu P. B. (2019). Ceram. Int..

[cit34] Dong X. W., Zhang Y. Y., Wang W. J., Zhao R. (2017). J. Alloys Compd..

[cit35] Han X., Yang Y. J., Zhou J. J., Ma Q. X., Tao K., Han L. (2018). Chem.–Eur. J..

