# Supplementary material for: Constructing high-performance electrode materials using core–shell ZnCo2O4@PPy nanowires for hybrid batteries and water splitting
Source: RSC Adv. 2020 Jul 29;10(47):28324–31. doi: 10.1039/d0ra05177b (PMC9055827; doi:10.1039/d0ra05177b)
Supplement: RA-010-D0RA05177B-s001 [file RA-010-D0RA05177B-s001.pdf]

Electronic Supplementary Material (ESI) for RSC Advances.
This journal is © The Royal Society of Chemistry 2020

Supporting Information

Constructing high performance electrode materials through core-shell $ZnCo_2O_4$@PPy nanowires for hybrid battery and water splitting

Xiaoyun Liu[1,*], Qian Li[1], Yanli Qin[1], Yueqiu Jiang[2,*],

[1]School of Science, Shenyang Ligong University, Shenyang 110159, P. R. China

[2] Department of Development and Planning, Shenyang Ligong University, Shenyang 110159, P. R. China

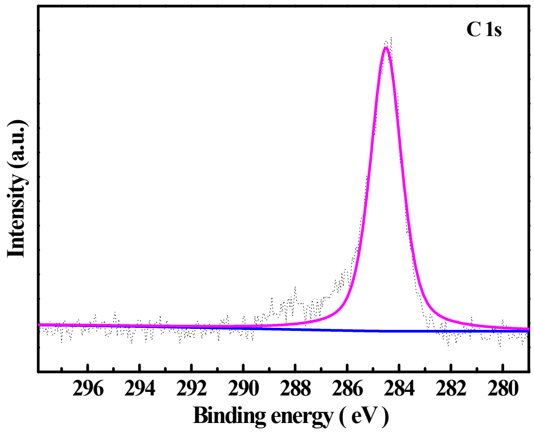

Fig. S1. C1s XPS spectra of $ZnCo_2O_4$@PPy-50 electrode material

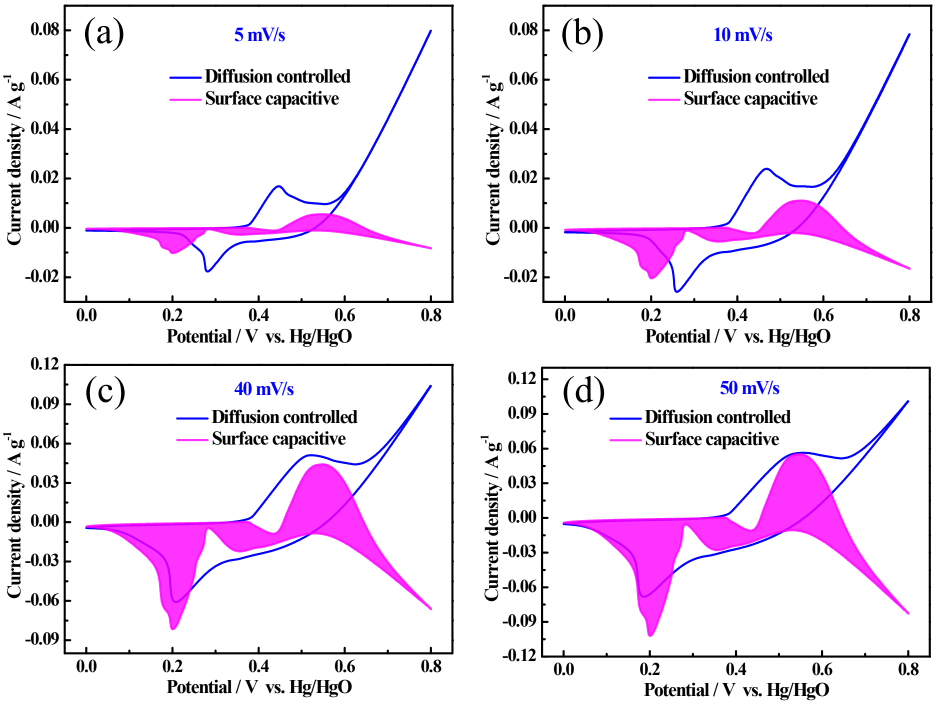

Fig. S2. Capacitive and diffusion contributions at a scan of 5, 10, 40 and 50 mV s^{-1}
